# A New Design of a Single-Device 3D Hall Sensor: Cross-Shaped 3D Hall Sensor

**DOI:** 10.3390/s18041065

**Published:** 2018-04-02

**Authors:** Wei Tang, Fei Lyu, Dunhui Wang, Hongbing Pan

**Affiliations:** 1 School of Physics, Nanjing University, Nanjing 210093, China; weitedit@outlook.com (W.T.); wangdh@nju.edu.cn (D.W.); 2 School of Electric Science & Engineering, Nanjing University, Nanjing 210093, China; lvfeieric@gmail.com

**Keywords:** three-contact Hall device, cross-shaped 3D Hall device, sensitivity, P-type layers

## Abstract

In this paper, a new single-device three-dimensional (3D) Hall sensor called a cross-shaped 3D Hall device is designed based on the five-contact vertical Hall device. Some of the device parameters are based on 0.18 μm BCDlite^TM^ technology provided by GLOBALFOUNDRIES. Two-dimensional (2D) and 3D finite element models implemented in COMSOL are applied to understand the device behavior under a constant magnetic field. Besides this, the influence of the sensing contacts, active region’s depth, and P-type layers are taken into account by analyzing the distribution of the voltage along the top edge and the current density inside the devices. Due to the short-circuiting effect, the sensing contacts lead to degradation in sensitivities. The P-type layers and a deeper active region in turn are responsible for the improvement of sensitivities. To distinguish the P-type layer from the active region which plays the dominant role in reducing the short-circuiting effect, the current-related sensitivity of the top edge (*S_top_*) is defined. It is found that the short-circuiting effect fades as the depth of the active region grows. Despite the P-type layers, the behavior changes a little. When the depth of the active region is 7 μm and the thickness of the P-type layers is 3 μm, the sensitivities in the *x*, *y*, and *z* directions can reach 91.70 V/AT, 92.36 V/AT, and 87.10 V/AT, respectively.

## 1. Introduction

In recent years, Hall sensors based on CMOS (Complementary Metal-Oxide-Semiconductor Transistor) technology have been widely used in manufacturing, electronics, aerospace, and other fields due to their low cost, high integration, and reliability. Meanwhile, in the field of scientific research, Hall sensors as an effective means of converting a magnetic field into an electrical signal are also attracting more and more attention [1,2,3,4].

In order to realize the measurement of a magnetic field in the *x*, *y*, and *z* directions, multiple horizontal Hall devices are placed in different directions [5,6]. However, the drawbacks of this method are the complicated package and the high cost. To solve these problems, horizontal and vertical Hall devices were integrated into the same chip [7,8,9,10,11,12]. Besides this, in virtue of different biases, a single device can be also used for the measurement [13,14,15,16]. Current-related sensitivity (*S_I_*), a main performance parameter of Hall sensors, is related to the doping concentration of the active region and the thickness parallel to the magnetic field. Meanwhile, adding P-type layers will allow sensitivity promotions for Hall sensors [4]. Due to the presence of the P-type layers, PN junctions are formed between the P-type and N-type regions so that the current near the top edge is compelled to flow along the PN junctions. This brings about positive effects for the sensitivities. Since the magnetic field components *Bx*, *By*, and *Bz* are perpendicular to each other, the requirements for the thickness of the active region are distinct. In addition, the performance of single-device 3D Hall sensors cannot be optimized simultaneously in all three directions. Therefore, it is essential to select a suitable technology parameter to maintain balance for the entire device. The finite element method (FEM) divides a problem or structure into smaller, simpler parts and make it easier to solve the problem. COMSOL is a FEM simulation software in which the geometric and process parameters of Hall sensors can be easily varied for further analysis. The 2D and 3D models can be chosen from diverse modules to perform the studies, such as AC/DC and semiconductor modules [17,18,19]. Besides this, the relationships between the temperature and sensitivities can be investigated by considering the temperature coefficient [17,20]. COMSOL simulation results can also be used to compare with the Verilog-A model implemented in Cadence [21]. The variety of functions in COMSOL provides an easy-to-use method for the performance prediction of Hall sensors.

This work designs a new three-contact vertical Hall device which derives from the five-contact vertical Hall device [22,23]. Three-contact vertical Hall devices have served as vertical Hall Magnetic Sensors, such as the four-folded three-contact structure. Compared with the five-contact, four-contact, and six-contact vertical Hall devices, the four-folded three-contact can be compatible with various offset cancellation techniques for horizontal Hall devices [24,25,26]. Herein, two orthogonal three-contact vertical Hall devices are integrated to construct a cross-shaped 3D Hall sensor which can be used to measure the magnetic field in 3D space by different biases. The aim of this work is to discuss the behavior of the cross-shaped 3D Hall sensor through an analysis of FEM calculations. Some of the device parameters are based on 0.18 μm BCDlite^TM^ technology provided by GLOBALFOUNDRIES. Section 2 introduces the shift from a five-contact to a three-contact vertical Hall device and the basic principle of the three-contact device for measuring the magnetic field parallel to the device. Section 3 studies the cross-shaped 3D Hall device composed of two cross-like three-contact vertical Hall devices. Section 4 investigates the respective influences of the active region and P-type layers on the current-related sensitivity. Section 5 summarizes all of the work and presents conclusions.

## 2. Five-Contact to Three-Contact Vertical Hall Device

**Figure 1:**
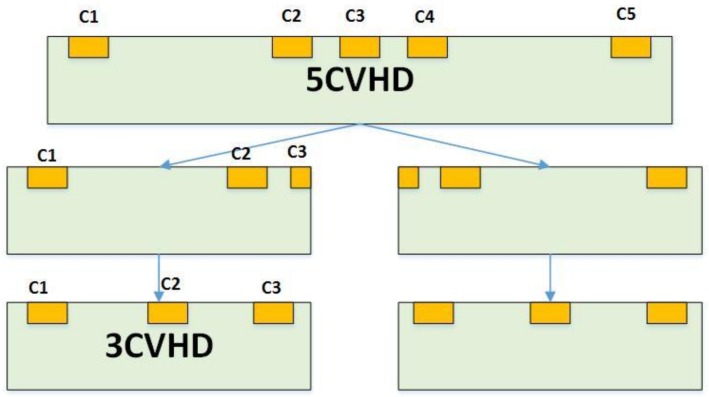
A shift from five-contact to three-contact vertical Hall device. The light-green areas represent the active regions and the yellow ones represent the contacts.

Figure 1 depicts the shift from a five-contact to a three-contact vertical Hall device. The five-contact vertical Hall device is sensitive to the magnetic field parallel to the device plane. C1, C3, and C5 are the biasing contacts, while C2 and C4 are the sensing contacts. Two orthogonal five-contact vertical Hall devices in a cross shape can be used to measure the *x* and *y* components of the magnetic field. If C2, C3, and C4 are ignored, the device can serve as a cross-like horizontal Hall device. However, the measuring method is fairly complex, and the presence of C2, C3, and C4 contacts will cause a severe short-circuiting effect. This because the doping concentration at the contacts is higher than that at the active region. The conductivity of an isotropic and homogeneous conductor is given by

(1) σ=nqμ

where *σ* represents the conductivity, *n* represents the carrier densities, and *μ* represents carrier mobility. It is easy to find that the conductivity increases with the doping concentration, even though the carrier mobility declines. In COMSOL, the material of the contacts is set as copper. Due to the higher conductivity at the contacts, the biasing current tends to flow to these areas, which would cause a short-circuiting effect. Converting a five-contact to a three-contact vertical Hall device can reduce the short-circuiting effect as well as the complexity by decreasing the number of contacts.

To make up a three-contact vertical Hall device, the five-contact device is divided into two halves and C2 is placed in the middle of the device. Then, a new contact C3 is placed at the symmetrical position of C1 with respect to C2 to construct a new device. The new device is called a three-contact vertical Hall device. Table 1 lists the comparisons between five-contact and three-contact vertical Hall devices, where the characteristics are physical symbols instead of detailed information. Because the three-contact vertical Hall device directly halves the length of the five-contact vertical Hall device, this will cause double power consumption of the device when biased by identical currents. To detect the magnetic field parallel to it, the three-contact vertical Hall device needs to be applied using opposite biases. Thus, the recognition rate is limited compared with the five-contact vertical Hall device. When biased by reverse currents, the deflection of the moving charge carriers due to the magnetic field is opposite. A potential difference at C2 is the Hall voltage of the three-contact vertical Hall device.

**Figure 2:**
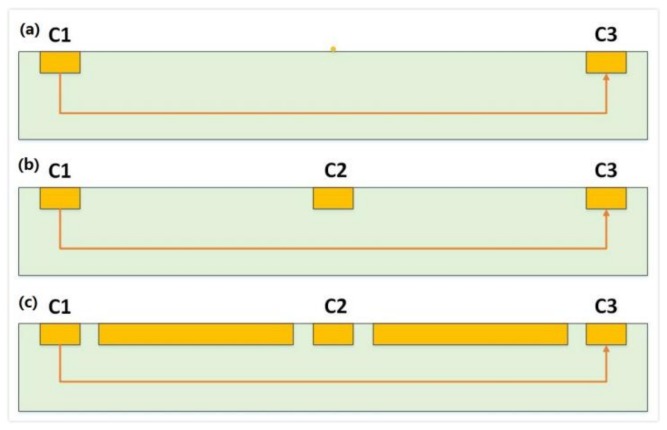
(**a**) Three-contact vertical Hall device without sensing contact. (**b**) Three-contact vertical Hall device with sensing contact and (**c**) three-contact vertical Hall device with sensing contact and P-type layers.

**Figure 3:**
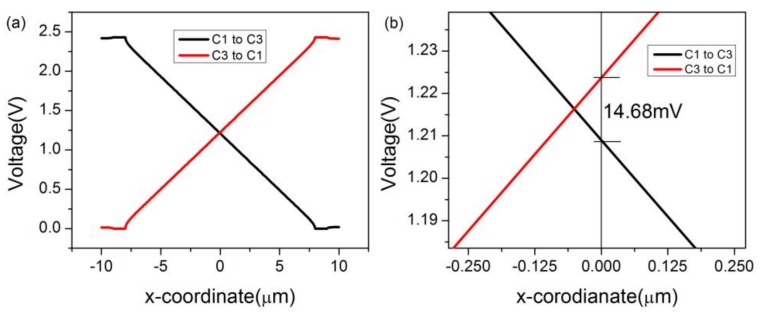
(**a**) Voltage along the top edge of the three-contact vertical Hall device without C2 and (**b**) distribution of voltage around the middle of the device under different biases.

**Figure 4:**
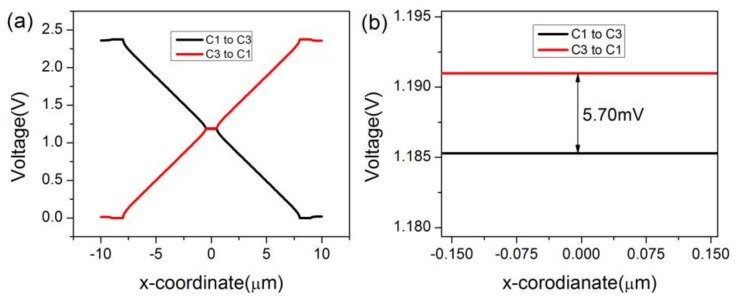
(**a**) Voltage along the top edge of the three-contact vertical Hall device with C2 and (**b**) distribution of voltage around C2 under different biases.

In COMSOL, the magnetic field is set as a constant, 1T. A 2D model is preferred over a 3D model for the ease of constructing a three-contact vertical Hall device. Meanwhile, C1, C2, and C3 are set as the Terminal, Floating Potential, and Ground, respectively. The biasing current is applied to the three-contact vertical Hall device via the biasing contacts-C1 and C3. For the purpose of investigating the influence of sensing contact on the behavior of the three-contact vertical Hall device, a point-like sensing contact (without C2) is placed at the center of the device, and then a 1 μm × 3 μm × 0.133 μm sensing contact C2 takes up this position. The two different structures are depicted in Figure 2a,b, respectively. Main simulation parameters are listed in Table 2. Figure 3a shows the voltage along the top edge of the device under different biases and 3b shows the distribution of voltage near C2. If the sensing contact is a point or neglected, the device’s Hall voltage is 14.68 mV. When the sensing contact is present, as shown in Figure 4, the device’s Hall voltage falls from 14.68 mV to 5.70 mV. This loss demonstrates a direct link between the sensing contact and sensitivities.

**Figure 5:**
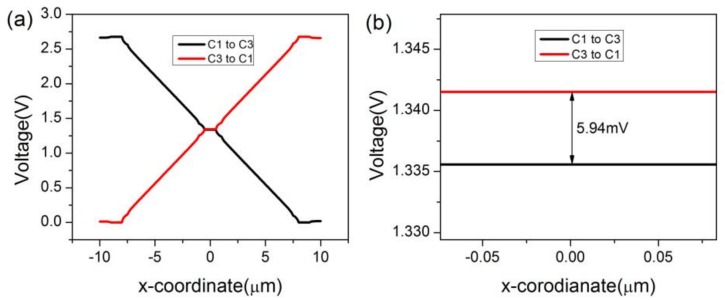
(**a**) Voltage along the top edge of the three-contact vertical Hall device with C2 and P-type layers and (**b**) distribution of voltage around C2 under different biases.

**Figure 6:**
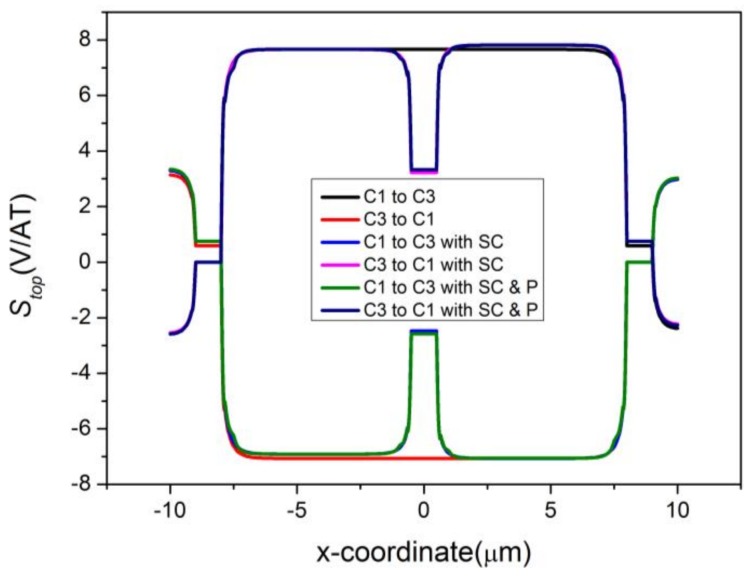
Effects of sensing contact and P-type layers on *S_top_*. SC represents the sensing contact and P represents the P-type layers.

For further improvement of the sensitivities, P-type layers described in Figure 2c are put between C1 (C3) and C2. In COMSOL, the P-type layers can be simply set to a material with zero conductivity to imitate the hindrance of the PN junctions to the biasing current. Judging from Figure 4 and Figure 5, the Hall voltage augments from 5.70 mV to 5.94 mV with an increasing rate of 4.2%. This indicates that an extra P-type layer can fortify sensors against the short-circuiting effect. To get a whole picture of the three different structures, the current-related sensitivity of the top edge, *S_top_*, is defined as shown below:

(2) $$S_{top}=\frac{V_{top}(B\neq 0)-V_{top}(B=0)}{IB}$$

where the *V_top_* is voltage along the top edge. *S_top_* can be utilized to distinguish a P-layer from the active region to see which plays the dominant role in suppressing and weakening the short-circuiting effect. With *S_top_*, the current-related sensitivity of the three-contact vertical Hall device is given by

(3) $$S_{I}=|S_{top}(C1\;\to \;C3)-S_{top}(C3\;\to \;C1)|$$

where *C*1 → *C*3 and *C*3 → *C*1 represent the reverse biases. The changing curves of *S_top_* are shown in Figure 6. In comparison with Figure 3, Figure 4 and Figure 5, the sensitivity change can now be calculated immediately on the basis of Figure 6. The absolute value of *S_top_* has a swift fall at C2, corresponding to the great loss in *S_I_*. Besides this, *S_top_* increases slightly due to the presence of P-type layers.

**Figure 7:**
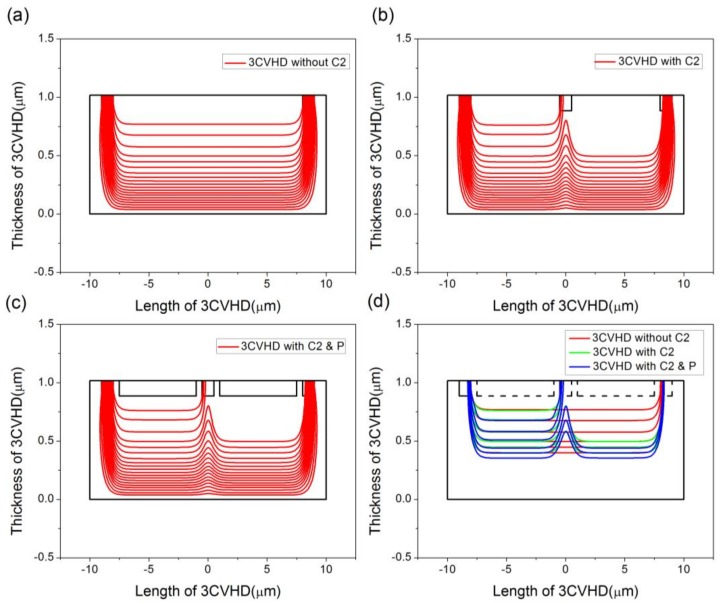
Effects of sensing contact and P-type layers on distribution of current density. (**a**) Sensor without C2. (**b**) Sensor with C2. (**c**) Sensor with C2 and P-type layers and (**d**) comparisons between three structures. The device is 1.018 μm deep and P-type layers are 0.133 μm deep.

**Figure 8:**
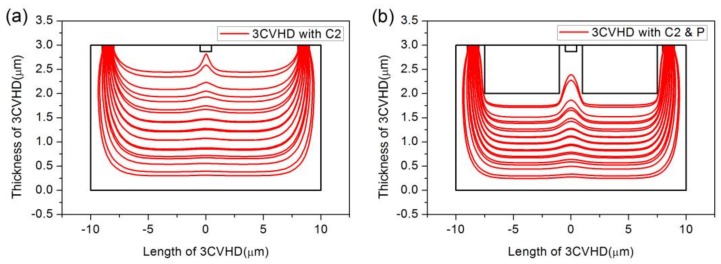
Comparisons in the distribution of current density. (**a**) Sensor with C2. (**b**) Sensor with C2 and P-type layers. The device is 3 μm deep and P-type layers are 1 μm deep.

The impacts of the sensing contact and P-type layers are both taken into account by inspecting the distribution of the current density. Figure 7 shows the effects of sensing contacts and P-type layers on the distribution of current density when the device is 1.018 μm deep and the P-type layers are 0.133 μm deep. Figure 7d selects some lines of current density near the top edge for more precise comparisons. Comparing Figure 7b,c with Figure 7a, we can find that the short-circuiting effect caused by the sensing contact prevails. In addition, the current is forced to flow along the boundaries of the P-type layer, as shown in Figure 7c, to reduce the effect. We infer that if a current can flow into the deep part of the Hall sensors, the behavior of the biasing current will be restrained. It is then expected that a device with intermediate depth and adjustable P-type layers may contribute to short-circuiting effect reduction. To further check this hypothesis, we study the comparisons in the distribution of the current density when the device is 3 μm deep and the P-type layers are 1 μm deep, which is shown in Figure 8. The biasing current stays away from the C2 contact compared with the device with depth 1.018 μm, even though there are no P-type layers. Meanwhile, it is obvious that the behavior will be better when the P-type layers are existent. Through an analysis of FEM calculations in COMSOL, it turns out that the current-related sensitivity rises by 18.8%, from 11.22 V/AT to 13.33 V/AT.

**Figure 9:**
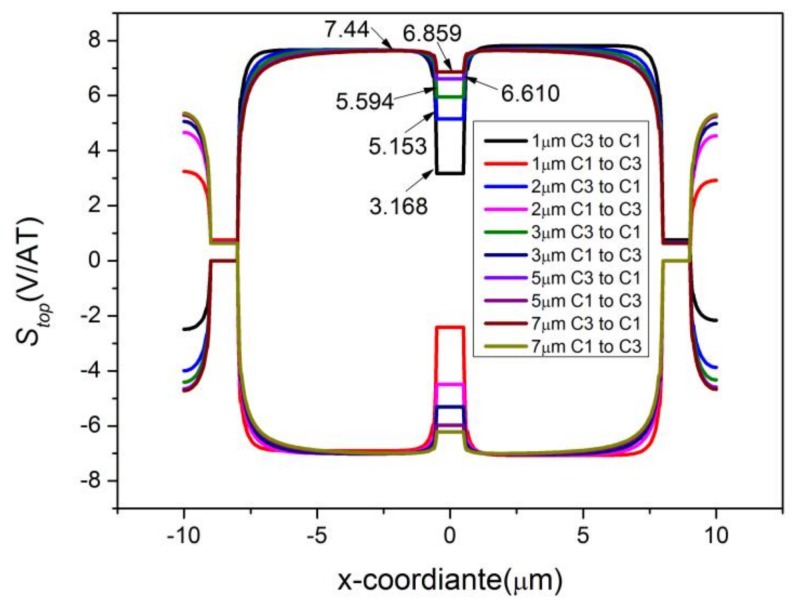
Comparisons between different depths of the active region in *S_top_*. The depth changes from 1 μm to 7 μm.

Figure 9 shows the current-related sensitivity of the top edge at different depths. It is justified that the deeper active region itself can cause degradation of the short-circuiting effect by preventing the biasing current from getting close to the sensing contact and making the voltage along the bottom edge become constant to compensate for the wasted Hall voltage [27]. We can even believe that if there is no sensing contact, the depth of the active area would have no effect on the sensitivities.

## 3. Performance of the Cross-Shaped 3D Hall Device

**Figure 10:**
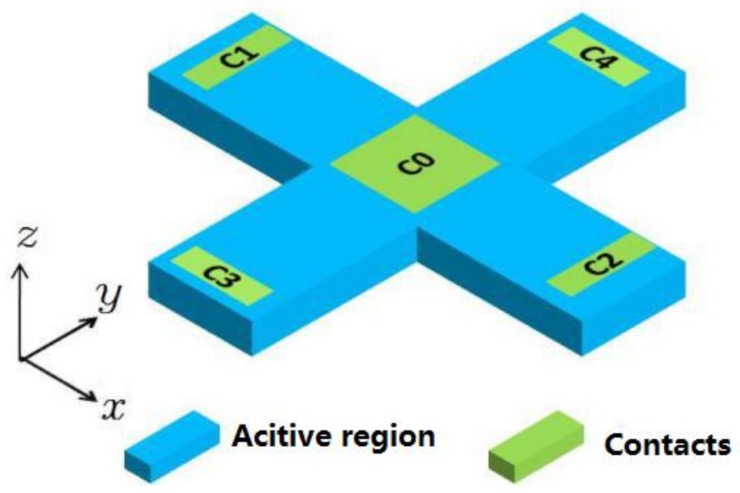
Diagram of the cross-shaped 3D Hall device. Two orthogonal three-contact vertical Hall devices are placed in a cross shape.

To accommodate measurement of the magnetic field in 3D space, two three-contact vertical Hall devices are placed orthogonally. Each of them can be used to detect the components *Bx* and *By*. When ignoring the sensing contact, the device can be regarded as a horizontal Hall device to measure *Bz*. The cross-shaped 3D Hall device is equivalent to the horizontal Hall device with an extra contact C0, which is depicted in Figure 10. Table 3 lists the main simulation parameters of the cross-shaped Hall device.

**Figure 11:**
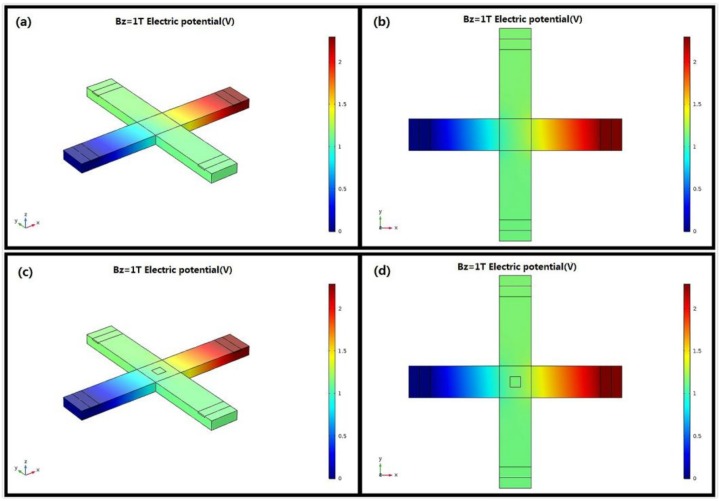
Voltage distribution of the horizontal and cross-shaped 3D Hall devices in 3D space and in the *x*, *y* plane. (**a**,**b**) Horizontal Hall device. (**c**,**d**) Cross-shaped 3D Hall device.

**Figure 12:**
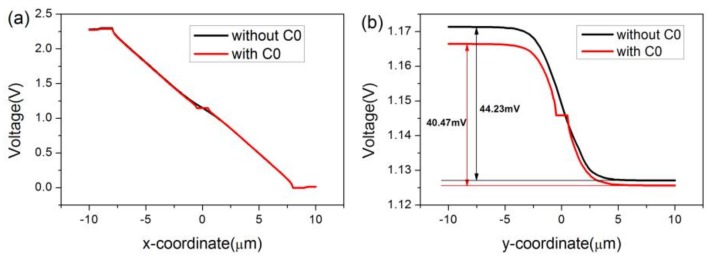
Effects of sensing contact on the distribution of voltage in *x* and *y* directions. (**a**) Horizontal Hall device and (**b**) cross-shaped 3D Hall device.

To discuss the differences between the horizontal Hall device and the cross-shaped 3D Hall device, *Bx* and *By* are set to 0 and *Bz* is set to 1T. Figure 11 depicts the voltage distribution of the Hall devices in the 3D space and *x*, *y* plane for developing a sense of the whole. The horizontal Hall device is on the top, and the cross-shaped 3D Hall device is on the bottom. Despite the introduction of the sensing contact C0, the voltage distribution of the device in all directions shows almost no change. Hence, the cross-shaped 3D Hall devices can be employed as horizontal sensors. However, due to the short-circuiting effect caused by C0, the current-related sensitivity in the *z* direction alters from 44.23 V/AT to 40.47 V/AT, as shown in Figure 12.

**Figure 13:**
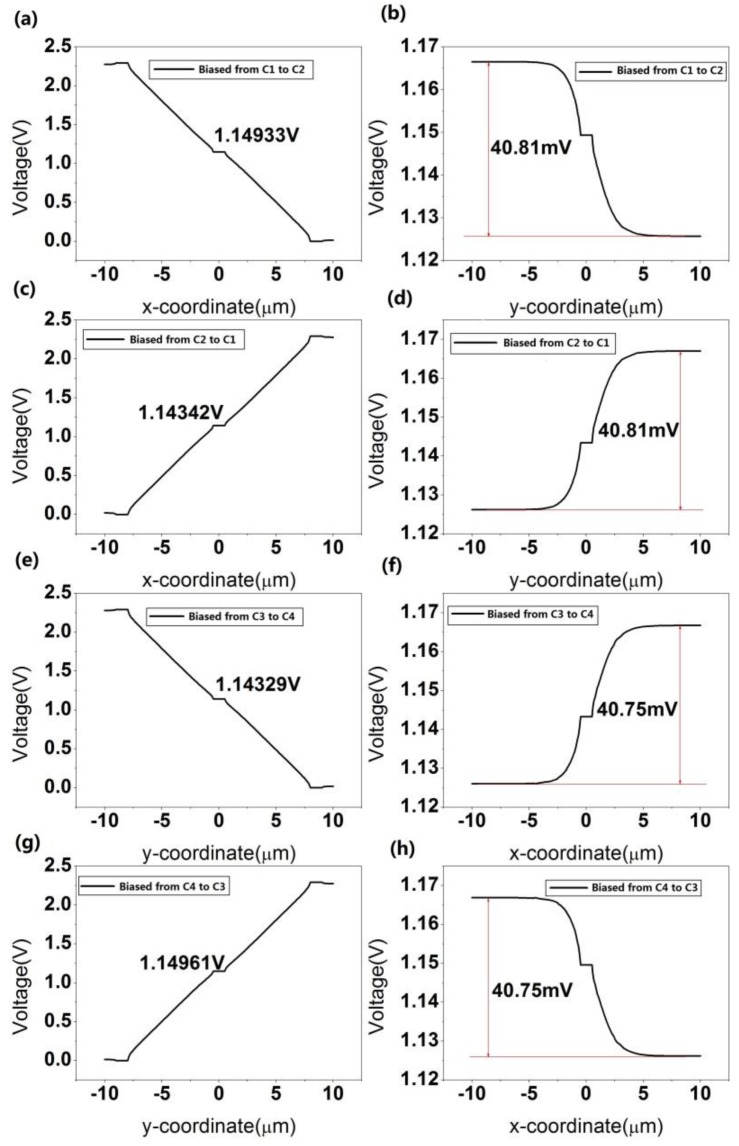
Simulation for cross-shaped 3D Hall device under different biases. (**a**,**b**) Distribution of voltage in *x* and *y* directions when biased from C1 to C2, respectively. (**c**,**d**) Distribution of voltage in *x* and *y* directions when biased from C2 to C1, respectively. (**e**,**f**) Distribution of voltage in *y* and *x* directions when biased from C3 to C4, respectively. (**g**,**h**) Distribution of voltage in *y* and *x* directions when biased from C4 to C3, respectively.

For details of the cross-shaped 3D Hall device, the magnetic field is set to 1T in all three directions. Figure 13 shows the simulation results of the cross-shaped 3D Hall device under different biases and directions. It can be calculated that the current-related sensitivity in the *x* direction is 5.91 V/AT and the sensitivity in the *y* direction is 6.32 V/AT. When the biasing current is applied from C1 to C2 or C2 to C1, the *S_I_* in the *z* direction is 40.81 V/AT. The sensitivity can reach up to 40.75 V/AT if the bias is from C3 to C4 or C4 to C3.

There are some differences between the components of the sensitivities in the *x* and *y* directions. When the cross-shaped 3D Hall device is used as a horizontal device, there remain some differences in the sensitivities of the *z* direction under different biases. This is because COMSOL solves a complex problem by dividing it into smaller, simpler parts. The software uses the “Mesh” function to define the amount and size of the domain and boundary elements. During the simulation, a “Free Triangular Mesh” (simpler part) is selected. This method results in the components of the cross-shaped 3D Hall device in the *x* and *y* directions being not identical as well as in the small discrepancies in the sensitivities.

Furthermore, the sensitivity of the device in the *z* direction is much better than those in the *x* and *y* directions when the active region is 1.018 μm. This originates from the different demands for the depth of the active region in the three directions. The shallow depth limits the flow of current, but increases the Hall voltage of the *z* direction. To overcome this difficulty, a CMOS technology with an additional deep *n*-well is compatible with the requirements for high sensitivities in the *x* and *y* directions.

## 4. Improvement of the Cross-Shaped 3D Hall Device

The doping concentration and the depth of the active region can affect the behavior of the Hall sensors. Reducing the doping concentration of the active region can result in a promotion of the current-related sensitivity of the Hall sensors, mainly due to the increase of the Hall coefficient. In this section, the doping concentration is lower than the one in the standard process and dramatically enhances the sensitivities. The conventional Hall device requires a shallow depth of the active region, which is beneficial to reducing the thickness along the magnetic field and promoting the increase of the Hall voltage and sensitivities. The vertical Hall device, however, requires a deep active region for a high sensitivity. Therefore, consideration and analysis of the sensitivities in the horizontal (*x* and *y*) and vertical (*z*) directions are needed.

**Figure 14:**
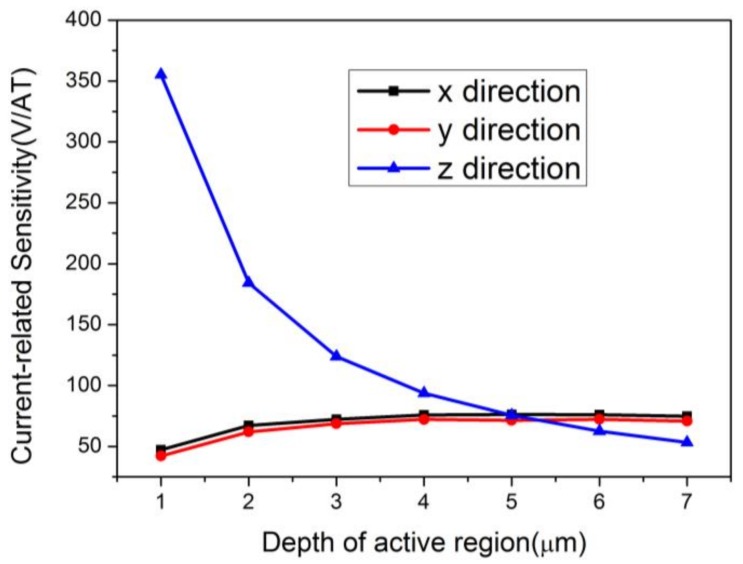
Relationships between the current-related sensitivities in three directions and the active region’s depth. The depth changes from 1 μm to 7 μm.

Figure 14 shows that the current-related sensitivity in the *z* direction is highly dependent on the depth of the active region and that the curve of sensitivity changing with the depth is similar to an inverse proportional function. In addition, sensitivities in the *x* and *y* directions show little difference, with both showing a similar slightly increasing tendency. These simulation results indicate that 5 μm is an optimized depth for balancing the performance in all directions. The sensitivities in the directions of *x*, *y*, and *z* are 76.19 V/AT, 71.50 V/AT, and 75.70 V/AT, respectively.

**Figure 15:**
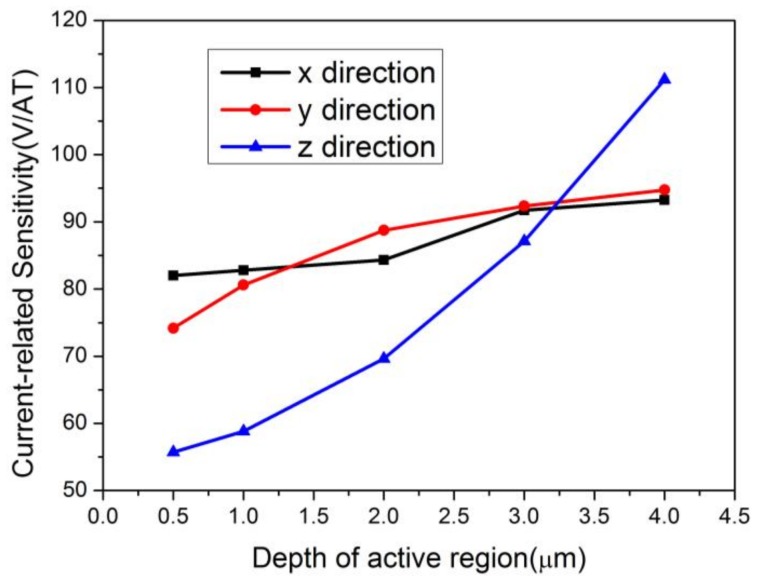
Relationships between the current-related sensitivities in three directions and the P-type layers’ depth. The depth of the active region is 7 μm.

**Figure 16:**
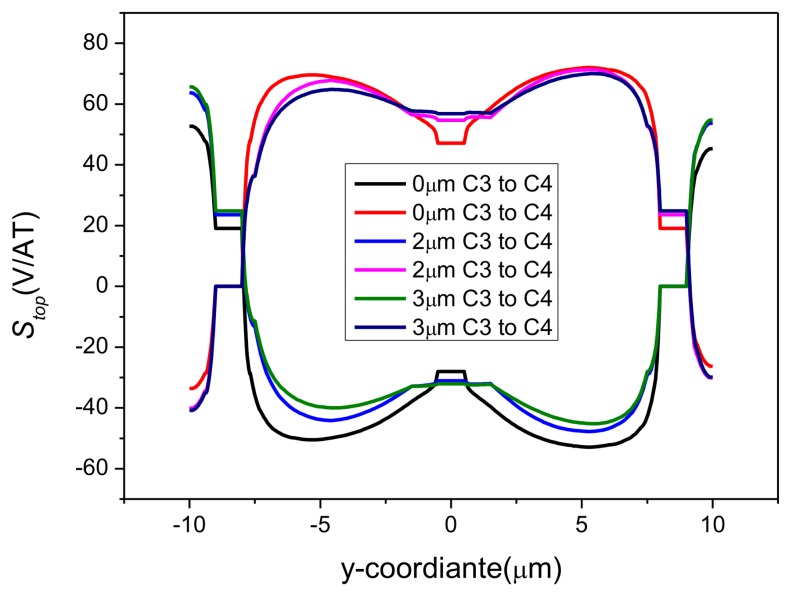
Comparisons between different depths of P-type layers in *S_top_*. The cross-shaped 3D Hall device is 7 μm deep. ‘0 μm’ represents a device without P-type layers.

The effects of P-type layers with various thickness on the sensitivities of the cross-shaped 3D Hall device were also studied. The depth of the active region was set to 7 μm for a full investigation. Figure 15 shows the current-related sensitivities in the *x*, *y*, and *z* directions with respect to the depth of the P-type layers. Sensitivity in the *z* direction is affected obviously with the increase of the P-type layers’ thickness for degradation in the depth along *Bz*. The components of sensitivities in the *x* and *y* directions show a small increasing response to the change of P-type layers. Furthermore, the differences between the two components vary irregularly. The differences also originate from the simpler parts divided by the Mesh function. To gain a better sense of the P-type layers’ effects on the horizontal direction, we depicted the changing curve of *S_top_* corresponding to the depth as shown in Figure 16. It is found from the comparison of Figure 9 and Figure 16 that the short-circuiting effect has faded since the active region is 7 μm. Despite the P-type layers, the behavior changes a little. The sensitivities balance when the depth of the P-type layers is 3 μm and reach up to 91.70 V/AT, 92.36 V/AT, and 87.10 V/AT in the directions of *x*, *y*, and *z*, respectively.

To provide a better comparison, we list some key prior works. Those works which focused on integrating horizontal and vertical Hall devices into the same chip have been excluded. Detailed information is displayed in Table 4. The sensitivities in the horizontal and vertical directions cannot be optimized simultaneously due to the opposite demands on the active region [13]. Though a balanced performance is achieved [14,15,16], there is a compromise in one of sensitivities in the horizontal and vertical directions. In our work, the sensitivities of *x*, *y*, and *z* can reach 91.70 V/AT, 92.36 V/AT, and 87.10V/AT by adjusting the depth of the active region and P-type layers, albeit in COMSOL. This can provide another method for the study of the single-device 3D Hall sensors.

## 5. Conclusions

In order to measure the magnetic field in three directions, we designed a new cross-shaped 3D Hall device by integrating two three-contact vertical Hall devices derived from the five-contact vertical Hall device. Some of the device’s parameters were based on 0.18 μm BCDlite^TM^ technology provided by GLOBALFOUNDRIES. The behavior was studied through an analysis of FEM calculations. A fitting model in COMSOL can provide more chances for exploration and thus reduce the number of iterations and improve efficiency before manufacturing devices. To have detailed knowledge of the devices, the sensing contact and P-type layers were taken into account by analyzing the distribution of the voltage along the top edge and the current density. The P-type layers were demonstrated to be able to weaken the short-circuiting effect by keeping the behavior of the current under control. Meanwhile, adjusting the depth of the active region and the thickness of the P-type layer provides a way to balance the performance. When the depth of the active region and the thickness of the P-type layer are set to 7 μm and 3 μm, the sensitivities in the directions of *x*, *y*, and *z* are 91.70 V/AT, 92.36 V/AT, and 87.10 V/AT, respectively. In general, FEM provides an easy-to-use method for breaking down a complex problem into a simple one and for the preverification of the performance of devices.

## Figures and Tables

**Table 1:** Comparisons between five-contact and three-contact vertical Hall devices. The characteristics of the devices are physical symbols instead of detailed information. *f* represents the frequency of conversion of the biasing current.

Parameter	Five-Contact Vertical Hall Device	Three-Contact Vertical Hall Device
Length	*l*	*l/2*
Width	*w*	*w*
Thickness	*t*	*t*
Biasing current	*I*	*I*
Power	*P*	*2P*
Recognition rate	*Real-time*	*1/f*

**Table 2:** Three-contact Hall device parameters and operating characteristics.

Parameter	Symbol	Value	Unit
Sensor length	*l*	20	μm
Sensor width	*w*	3	μm
Sensor thickness	*t*	1.018	μm
Contact length	*l_c_*	1	μm
Contact width	*w_c_*	3	μm
Contact thickness	*t_c_*	0.133	μm
P-type layer thickness	*t_p_*	0.133	μm
Biasing current	*I*	1	mA

**Table 3:** Cross-shaped 3D Hall device parameters and operating characteristics.

Parameter	Symbol	Value	Unit
Sensor length	*l*	20	μm
Sensor width	*w*	3	μm
Sensor thickness	*t*	1.018	μm
C0/C1/C2/C3/C4 length	*l_c_*	1	μm
C1/C2/C3/C4 width	*w_c_*	3	μm
C0 width	*w_c0_*	1	μm
C0/C1/C2/C3/C4 thickness	*t_c_*	0.133	μm
P-type layer thickness	*t_p_*	0.133	μm
Biasing current	*I*	1	mA

**Table 4:** Performances of the single-device 3D Hall sensors based on different technologies.

Time	Technology	Sensitivity in Vertical Direction (V/AT)	Sensitivity in Horizontal Direction (V/AT)	Reference
2003	Silicon Technology, with Low Doped N–Si Substrate	29	85	[13]
2009	CMOS Technology	36	27	[14]
2015	Special Silicon Technology	33.3	33/33.9	[15,16]
